# FILM: Filtering and Machine Learning for Malware Detection in Edge Computing

**DOI:** 10.3390/s22062150

**Published:** 2022-03-10

**Authors:** Young Jae Kim, Chan-Hyeok Park, MyungKeun Yoon

**Affiliations:** 1 Common Computer, 8, Maeheon-ro, Seocho-gu, Seoul 06797, Korea; byeongal@kookmin.ac.kr; 2 Department of Computer Science, Kookmin University, 77, Jeongneung-ro, Seongbuk-gu, Seoul 02707, Korea; pbkpch@kookmin.ac.kr

**Keywords:** malware detection, edge computing, machine learning, cyber security

## Abstract

Machine learning with static-analysis features extracted from malware files has been adopted to detect malware variants, which is desirable for resource-constrained edge computing and Internet-of-Things devices with sensors; however, this learned model suffers from a misclassification problem because some malicious files have almost the same static-analysis features as benign ones. In this paper, we present a new detection method for edge computing that can utilize existing machine learning models to classify a suspicious file into either benign, malicious, or unpredictable categories while existing models make only a binary decision of either benign or malicious. The new method can utilize any existing deep learning models developed for malware detection after appending a simple sigmoid function to the models. When interpreting the sigmoid value during the testing phase, the new method determines if the model is confident about its prediction; therefore, the new method can take only the prediction of high accuracy, which reduces incorrect predictions on ambiguous static-analysis features. Through experiments on real malware datasets, we confirm that the new scheme significantly enhances the accuracy, precision, and recall of existing deep learning models. For example, the accuracy is enhanced from 0.96 to 0.99, while some files are classified as unpredictable that can be entrusted to the cloud for further dynamic or human analysis.

## 1. Introduction

The number of malicious software, malware, has been increasing rapidly. More than one billion unique malware samples were collected in 2014, and Virustotal collects more than one million suspicious files every day in 2020 [1,2]. Recent studies show that similar malware files are frequently observed in large malware datasets [1,3]; therefore, anti-malware companies have started augmenting their malware detection with Machine Learning (ML) that can detect a previously unseen but similar malware samples [4,5,6].

The rapid development of the Internet-of-Things (IoT) and smart devices have been incentivizing the advancement of edge computing [7]. Edge computing can overcome certain limitations of the cloud computing, such as service delays, network disconnections, and privacy problems, by hosting computation tasks close to the data sources and end users. Recently, edge intelligence attracts significant attention for facilitating the deployment of deep learning services by using edge computing [8].

A malware attack is one of the most critical threats to edge computing and cyber security [7]. Recent malware can evade simple signature-matching detection; therefore, anti-malware solutions adopt ML techniques to detect unseen malicious files. An edge node is a good place to provide ML-based malware detection services. First, when IoT devices do not have enough computing resources, edge nodes can protect them as a secure gateway. Second, an edge node can select and provide an appropriate level of malware detection services for IoT devices. Malware can be detected by simple signature matching, static analysis, dynamic analysis, and expensive human expert analysis. Signature matching or static analysis services can be provided by an edge node, but highly complicated and evasive malware should be entrusted to the cloud for detailed analysis. Third, an edge node can provide malware analysis service without interruption even when the cloud or internet is not stable. Finally, privacy and data leakage problems can be mitigated because most of suspicious files are processed within the internal edge nodes rather than public clouds.

Although edge computing is a promising security option for IoT, there are challenging problems in ML-based malware detection. Many researchers have extensively studied ML-based malware detection as a binary classification problem to distinguish malicious files from benign ones [1,3,4,6,9,10]; therefore, ML models always classify files as benign or malicious. However, some malware files cannot be detected by the ML models if proper features are not extracted from the files. For example, malware files of an installer type cannot be discerned from benign installer files when only static analysis features are used for ML training; however, current ML models are forced to classify a file into either a benign or malicious one. This problem becomes worse for edge computing. An edge node provides malware detection service even when the cloud is not available; therefore, the malware detection of edge computing should be more accurate and complete for the prediction. We argue that the ML models for edge computing should be able to not only decide if a suspicious file is benign or malicious, but also estimate how confident the decision is. If the edge node is not confident about the decision, it would be better for the node to declare the file as unpredictable. Then, the file can be uploaded to the cloud for further dynamic analysis or even entrusted to human experts.

In this paper, we present a new ML model for edge computing that can classify a suspicious file into three classes, benign, malicious, and unpredictable. If the file is unpredictable, the edge node uploads the file into the cloud for further analysis. When the file is predicted as either benign or malicious, the accuracy should be much higher than that of the state-of-the-art malware detectors. This new model is motivated by recent out-of-distribution studies in the literature of neural networks [11]. To the best of our knowledge, this is the first ML model that improves accuracy, precision, and recall by introducing unpredictability for the malware detection problem. The main contributions of this paper are as follows:

- We introduce an edge computing service of ML-based malware detection while considering different types of malware analysis methods and their costs, e.g., static, dynamic, and human analysis.
- We develop a new ML technique that can classify a suspicious file into three classes of benign, malicious, and unpredictable by using the result of a final sigmoid function from deep neural networks.
- Through experiments on real malware datasets, we show that the new scheme detects malicious files more accurately than the state-of-the-art methods. For example, the accuracy is enhanced from 0.96 to 0.99, while some files are classified as unpredictable.
- The new scheme can be used orthogonally with any deep learning model. We test four well-known models and confirm that the accuracy is significantly enhanced for all the cases without increasing any time and space complexity.

The rest of the paper is organized as follows. Section 2 is the related work. We introduce intelligent edge computing for malware detection in Section 3. We present new ML models in Section 4. Section 5 shows the experimental results, and Section 6 is the conclusion.

## 2. Related Work

Because the large number of malware files overwhelms human resources and computing power for decades [2], ML has been adopted by anti-malware companies to detect unseen malicious files [1,4,5,12,13,14]. Recent studies have shown that both benign and malicious files have strong similarities among themselves [1,3], which explains the high accuracy of recent ML-based malware detection schemes [3,9,15,16,17,18].

In general, malware files of three different types are featured in datasets for ML-based malware detection: Portable Executable (PE) files [1,5,16,17], Android Package Kit (APK) files [15], and document malware files of Portable Document Format (PDF) or office documents [2]. For ML algorithms, traditional decision trees, random forests, support vector machines, and recent deep learning algorithms are widely used for malware detection [6]. Although recent studies adopt more deep learning algorithms [9,16,17,18], this does not mean that deep learning always outperforms classical algorithms. For example, Aghakhani et al. show that the random forest is the best classifier in their experiments [5].

The great success of deep learning in image recognition and natural language processing motivates researchers to invent malware detection schemes on neural networks (NN) [6,9,16,17,18]. They use different features and neural network models, such as fully connected networks [9], Convolutional Neural Networks (CNN) [16], gated-CNN [17], a combination of CNN and Recurrent Neural Networks (RNN) [18], etc. Building an input feature vector for ML-based malware detection models is a critical task. There are two main analysis methods to excerpt features from benign and malicious files, static vs. dynamic. Static analysis features are relatively easy to extract and require less computing resources and time, such as file header fields, strings, and byte *n*-grams [5,6,15,17]. Dynamic analysis requires more computing resources, time, and sophisticated setting for analysis tools to execute malware files [6,18]; however, it is almost impossible to develop feature vectors that can perfectly reflect all malicious behaviors because attackers always invent a new evasion technique.

Although the accuracy of ML-based malware detection seems high, above 95% as in [9,15,16,18], recent studies reveal their weakness and limitations; the features from packed files are not rich enough for ML models to operate on unseen packers [9]. A malware packer is a tool to mask a malicious file into a packed file through encryption, compression, or deliberate changes of the file format. Zhu et al. show that the labels of anti-virus engines from VirusTotal can be unstable, which affects the ML models [10]. They recommend to aggregate labels from a number of engines. Pendlebury et al. show the high accuracy of Android malware classifiers is commonly inflated due to pervasive sources of experimental bias: spatial bias and temporal bias [15]. Yang et al. study the concept drift problem that the testing malware data distribution is shifting from the original training data over time, causing major failures to the deployed model [19]. The authors present a contrastive learning idea to solve the problem.

Edge computing helps to overcome the limitations of cloud computing. Edge intelligence aims to facilitate the deployment of deep learning services at edge nodes [8]. Security is essential for edge computing and malware infection is one of the most critical threats [7]. Edge computing can provide security services to IoT devices that do not have enough computing power, and ML-based malware detection is one of the most practical and necessary security services. The level of malware analysis is diverse because some malware files can be detected with static feature-based ML models whereas others require expensive dynamic analysis or human analysis; therefore, an edge node needs to determine whether a suspicious file can be detected by itself or whether the file should be uploaded to the cloud for further analysis, which has not been studied enough.

Table 1 summarizes selected related work in terms of ML models, datasets, features, goals, and prediction types where SVM and RF represent support vector machine and random forest, respectively. The new method can work with any neural-network-based models and answer benign, malicious, or unpredictable, which makes it unique and different from others.

## 3. Malware Detection and Edge Computing

### 3.1. Malware Detection and Machine Learning

A large number of malware files overwhelm human analysts. Only a few files can be manually analyzed and the detection rules become available; therefore, security industries have started augmenting their malware detection and classification using ML and deep learning [4,6]. In this paper, ML implies supervised learning on a large dataset of malicious and benign files, extensively studied in this literature [3,4,9]. For simplicity, we consider file-type malware, but our proposed schemes can be easily extended to fileless malware.

Malware files can be analyzed at different levels and Table 1 summarizes the four types; signature matching is the main method for detecting classic malware files—it catches a specific string or regular expression in the malware files; however, recent malware can evade signature matching. Next, static analysis examines malware without running it while dynamic analysis executes the malware in a controlled and monitored environment. Static analysis features can be extracted quickly within a few seconds without heavy computing resources. Dynamic analysis requires virtual machines or a sandbox environment to run the malware file, which takes several minutes or hours. For example, the default period of the cuckoo sandbox is three minutes [18]. Human analysis is the most expensive, which may take several hours or even days [1,3,4].

**Figure 1:**
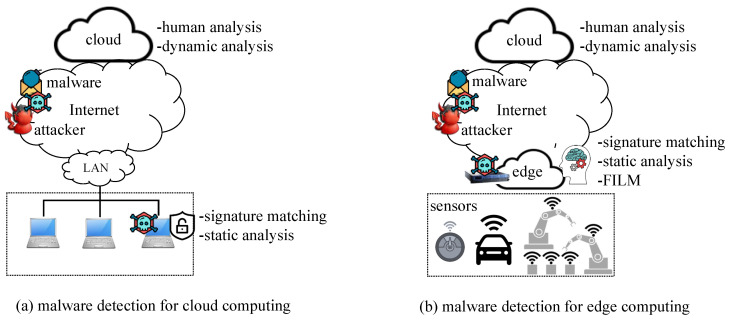
Comparison of machine-learning-based malware detection for (**a**) cloud computing and (**b**) edge computing.

After features from static or dynamic analysis are extracted, we can train ML models [9,16]. End-to-end deep learning can automate the feature extraction process [17]. Static features include strings, disassembled codes, file header information, and byte-stream distribution. Dynamic features include the sequence of application programming interface calls, mutex, and registry information [18]. Both the static and dynamic features have their own limitations. For example, the static features cannot distinguish the installer-type malware files from the benign installer files because their static features would be almost same. In this case, dynamic analysis should be applied to determine whether the file is malicious or benign [18]; however, the dynamic features may not contain enough information if the malware does not run to hide itself by recognizing the sandbox. This explains why malware analysis consists of multiple steps (see Table 2); for cloud computing environments, end devices such as laptops or PCs can detect malware files by signature matching and ML models on static features. A limited number of more complicated files can be uploaded to the cloud for further expensive dynamic or human analysis, as shown in Figure 1a.

Researchers have shown that ML-based malware detection with static features performs well with accuracy higher than 95% [3,4,9,15,16,17]. In this paper, we focus on ML-based malware detection using static features, but our idea can be applied to dynamic features as well.

### 3.2. Intelligent Edge Computing

The rapid development of IoT incentivizes the advance of edge computing that can overcome the cloud computing limitations, such as service delays, network disconnections, and privacy problems [7]. Edge computing is implemented by edge nodes that are located between the cloud and end devices, and generally deployed close to users. Edge intelligence attracts significant attention that aims to facilitate the deployment of deep learning services by edge computing. Vendors such as NVIDIA, Intel, and Samsung provide edge-computing hardware and systems that can provide a range of artificial intelligence applications [8].

In this paper, we present an intelligent malware detection scheme for edge or fog nodes. We assume that IoT devices with sensors do not have enough computing power to run ML models for malware detection, and a detection service is provided by the edge node. The edge node is a secure gateway that inspects the files downloaded from the internet to the end devices, as shown in Figure 1b. Some files may be malware that should be detected by the edge node. We assume that the edge node has an ML model that has been trained using static features. This assumption is practical because the edge nodes are not as powerful as the cloud. Most malware files can be detected by ML models using static analysis features that require only moderate computing resources. The edge node implements signature matching and ML-based static analysis. The cloud supports expensive dynamic and human expert analysis for those files that cannot be correctly analyzed at the edge node. When the connection between the cloud and an edge cannot be established, the edge can send the file to the fog server for analysis. In this case, the fog server should be available at the edge.

### 3.3. Problem and Motivation

Figure 1b shows edge computing where the ML model detects malware files using static analysis features at an edge node. A challenging problem is that current ML models categorize test files either as benign or malicious because the models are trained as binary classifiers [3,9,15,16,17]; however, some malware files and benign files have almost the same static analysis features; therefore, the models cannot correctly distinguish them. In this case, the models should provide a response as unpredictable rather than uncertainly predict either a malicious or benign label; then, the test file can be entrusted into the cloud for further analysis. We assume that a delayed analysis is more desirable than an incorrect prediction; however, those ML models that provide a benign, malicious, or unpredictable response have not been studied enough in the literature of malware detection at edge nodes.

A recent study reveals that we can estimate how confident ML models are when they predict the label of a test instance [11]. The authors present a baseline method that is useful for datasets of computer vision, natural language processing, and speech recognition. The idea is simple that we use the final softmax value from neural networks to determine how confident the model is for its prediction. Motivated by this method, we design a new ML-based malware detection scheme that can classify a suspicious file into three classes of benign, malicious, or unpredictable.

## 4. FILM: Filtering and Machine Learning for Malware Detection

We present a new ML-based malware detection scheme at edge nodes that can provide a response of benign, malicious, or unpredictable to a suspicious test file. We call this scheme Filtering and Machine Learning for Malware Detection (FILM) because the question about benign or malicious for a test file can be filtered out as unpredictable. The key idea is to use the score of the final activation function from neural networks as the index of how confident the ML is for its prediction, as in [11]. If the score is larger than a threshold, we accept the model prediction; otherwise, we consider the test file as unpredictable and that file can be uploaded to the cloud for further analysis. The prediction accuracy of FILM is enhanced while some files cannot be handled by edge nodes, which is the trade-off between accuracy and coverage.

### 4.1. Design Goals

A large number of unseen malware files are collected every day. ML-based malware detectors with static analysis features can correctly classify most of the files as benign or malicious [3,9,15,16,17]; however, some complicated files need to be handled by dynamic or human analysis, which requires more time and cost. These files should be classified as unpredictable by the static feature-based ML models; therefore, ML models should be able to classify a suspicious file as benign or malicious, and as predictable or unpredictable.

We design FILM to solve this challenging problem by using the prediction certainty of ML models. A recent study shows that the prediction certainty can be estimated by the value of the final activation function of neural networks [11]. To the best of our knowledge, FILM is the first ML model for edge nodes that can classify a test file into one of three categories, benign, malicious, and unpredictable. We take the value of the final activation function as a certainty score. If the score is larger than a threshold value, we take the prediction of the model that determines if the test file is either benign or malicious; otherwise, we do not trust the prediction and the final decision of the test file is entrusted to the cloud from the edge node.

**Figure 2:**
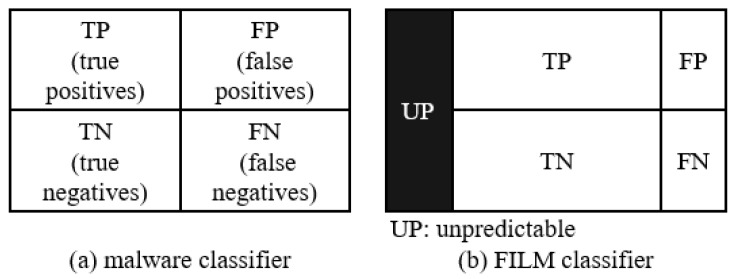
Comparison of prediction types, existing malware classifier vs. FILM. A new type of unpredictable appears in FILM.

Figure 2 shows the difference of prediction types between ordinary malware classifiers and FILM. The number of false classifications decrease in FILM whereas the number of unpredictable test files increase. There are four types of classification results for current malware detection:

- True Positive (TP): A malicious test file is classified as malicious.
- True Negative (TN): A benign test file is classified as benign.
- False Positive (FP): A benign test file is classified as malicious.
- False Negative (FN): A malicious test file is classified as benign.

Many ML-based malware detection schemes have been proposed and FILM can be orthogonally used with any model. In this paper, we implement FILM with four state-of-the-art models separately. Through experiments on real malware datasets, we confirm that FILM significantly alleviates the accuracy of all four models.

**Figure 3:**
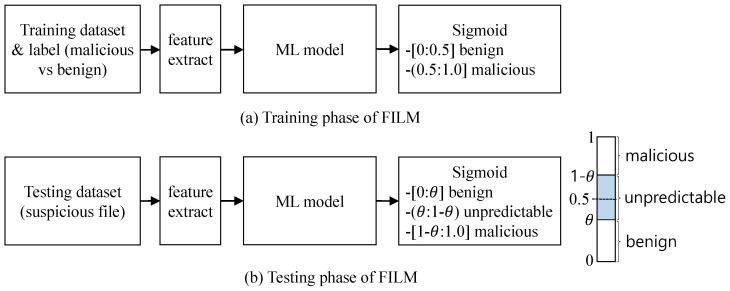
Training and testing phases of FILM. A sigmoid function is appended to an existing ML model. During the testing phase, the sigmoid value is interpreted in three ways.

The training and testing phases are shown in Figure 3. For a given ML model, we use its feature extraction module and the model as it is. We append a sigmoid function to the end of the model. When the sigmoid value is above 0.5, the model considers the file as malicious; otherwise, the model considers it as benign; therefore, the model is trained to generate the sigmoid value above 0.5 for a malicious file, and below 0.5 for a benign file during the training phase. For the testing phase, we interpret the sigmoid value differently; we consider that a sigmoid value of around 0.5 indicates that the model cannot exactly predict the label of a test file, which answers unpredictable instead of benign or malicious.

### 4.2. Training Phase

In this paper, we assume that the malware detection is a binary classification problem; a dataset of malware files and benign files with correct labels are available for supervised learning, as in [3,4,9,15,16,17]. The static analysis features are extracted from the dataset. Recent end-to-end deep learning models do not require an explicit feature extraction process because the features are learned during the training phase. During the training phase, an ML-based malware detection model is trained with a dataset with labels, as shown in Figure 3. In this paper, we use four different deep learning models as follows:

- Model 1 [9] is the first deep learning model for malware detection. It is a fully connected network with two hidden layers of 1024 input features or Parametric Rectified Linear Units (PReLU). The size of a feature vector is 1024 in total. The features include contextual bytes, PE import, string 2d histogram, and PE metadata, each of which makes 256 dimensions. The final output layer has one sigmoid unit. This is one of the most cited models in this literature [5,6].
- Model 2 [17] converts a binary file into a gray image file, and uses a deep convolutional neural network for malware family classification. Each byte in a binary file is interpreted as an unsigned integer in the range of 0∼255, and considered as a pixel. Through multiple convolutional layers and subsampling layers, the features of a binary file are extracted. The final fully connected layer classifies the binary file into one of nine malware families. We slightly modify this model to be a binary classifier for malware detection.
- Model 3 [16] is the first end-to-end deep learning model for malware detection. It reads raw byte sequences from a binary file for embedding and 1D gated-convolution that is also useful for a dynamic-feature model [18]. The byte sequence of a PE file is fed into the model. After zero-padding and embedding, 1d convolution and max-pooling layers are applied. Finally, the fully connected layer classifies the binary file as either benign or malicious.
- Model 4 [18] combine CNN and RNN using dynamic malware features. This model utilizes a feature hashing trick to encode the API call arguments associated with the API name [20]. The deep neural network architecture applies multiple gated-CNNs to transform the extracted features of each API call. The outputs are further processed through bidirectional RNN to learn the sequential correlation among API calls, and not only API names, but also API arguments. We modify the original model to process static analysis features of [5] to compare this model with other models.

We emphasize that FILM can be used together with any deep learning model for malware detection. We choose these four models because they are neural networks of a different architecture and well-known in this literature. The purpose of this paper is to present a new method to enhance the accuracy, precision, and recall of any model irrespective of the model’s architecture. The existing models only classify a suspicious file into a benign or malicious group while FILM can classify it into a benign, malicious, or unpredictable group. Those readers who are more interested in the details of each of the four models are referred to their original papers [9,16,17,18]. In the next section, we experimentally show that FILM significantly enhances the accuracy of all these models.

All four models have a sigmoid function as an output layer. This function produces a number between 0 and 1. We label a malware file as 1 and a benign file as 0. During the training phase, neural networks adjust their weights to be able to predict the correct class label of input samples. Actually, the training process of FILM is exactly the same as the original models.

Some readers may be interested in the time complexity and space complexity of FILM. We emphasize that the complexity depends on the original model itself not FILM. The result of the final sigmoid function in FILM is interpreted to decide if a given test file belongs to one of benign, malicious, or unpredictable class, which increases neither time nor space complexity.

### 4.3. Testing Phase

During the training phase, we trained neural networks to be a binary classifier that determines if a test file is benign or malicious. Current deep learning models consider a test file as malicious if the sigmoid value of the last layer is larger than 0.5; otherwise, the models consider the file as benign, which is the common strategy for neural networks in malware detection.

For the testing phase of FILM, we added a special step to estimate how confident the ML model is about their prediction. We adopt the main idea from [11], the performance of which is assessed against datasets in the literature of computer vision and natural language processing. To the best of our knowledge, FILM is the first that mitigates the problem of misclassified and out-of-distribution for malware detection.

Figure 3 illustrates how FILM classifies a test file into three categories of benign, malicious, and unpredictable. For a given test file, the output value of the sigmoid function, denoted as s, ranges from 0 to 1. We compute $s^{\prime }=\left|s-0.5\right|$, called the score of the test file. If the score is larger than a predefined threshold of θ, we accept the prediction of the model; otherwise, the test file is considered unpredictable, which means that this file cannot be correctly predicted by the model. We emphasize that the score of s’ becomes large if s is close to either 0 or 1. This idea is motivated by [11], and FILM is the first work to adopt the idea for malware detection. The difference is that FILM uses sigmoid as the final activation function. If the result of sigmoid is close to 1, we assume that the model is confident of its prediction that the test file would be malicious. If the sigmoid output is close to 0, we assume that the model strongly believe that the test file is benign. If the sigmoid output is around 0.5, we assume that the model is not certain about its prediction.

We believe that malware detection from static analysis features has limitations because some benign files and malware files have almost same static features. Analyzing our datasets, we observe that useful features from the headers of portable executable (PE) files are almost same between benign and malicious files of the installer type. Another observation is that the training accuracy does not become perfect even with a large number of epochs. We believe that static analysis features inherently have limitations for machine learning to achieve a perfect accuracy, which motivates us to develop FILM.

Through experiments on real malware and benign file datasets, we confirm that FILM significantly improves the prediction accuracy, compared to the state-of-the-art models of [9,16,17,18]. This implies that FILM can be applied to any neural network models for malware detection. Another advantage of FILM is that unpredictable cases are found by data-driven approaches not manual efforts. We emphasize that human resources are already scarce in analyzing sophisticated malware samples, and therefore the limitations of static analysis features should be found without additional human resources.

## 5. Experiments and Discussion

We experimentally evaluate FILM using datasets of more than 300,000 real malware and benign files. The deep neural networks of [9,16,17,18] are used as base models for malware detection. We compare the base models and FILM in terms of accuracy, precision, and recall. The experimental results confirm that FILM outperforms base models, but some files become unpredictable.

The dataset consists of real malware files and benign files of the PE file format. This file type has been popularly used for malware research [1,9]. We collected 177,600 malware files from the Virustotal website by using academic access [2]. In addition, we obtained 154,083 benign files from Windows systems in a university campus and from web crawling. The labels of the PE files are verified against the Virustotal reports.

We divided the dataset into two parts; 80% of the dataset is for training phase and the remaining 20% is for testing phase; therefore, the trained models do not see testing data at all during the training phase. For the training dataset, we again split it into four non-overlapping parts of the same size, and we conducted separate 4-fold cross validation experiments. During the cross validation of the training dataset, we adjusted an optimal value of θ for FILM, as shown in Figure 3. After the training phase finished, we used the testing dataset for the models to obtain the performance metrics of accuracy, precision, and recall.

We implement the deep neural network models of [9,16,17,18] separately as our base models. The static features are extracted by pefile, an open source tool developed by Python, as in [5]. Those who are interested in these models are referred to [9,16,17,18] for detailed information.

After the training phase completed, we used the test dataset to evaluate FILM and the base models. We use accuracy, precision, and recall to compare the performance. Accuracy is the ratio of the number of correctly predicted labels to the number of all test files. Precision is the number of correctly predicted malicious files to the number of files predicted as malicious. Recall is the number of correctly predicted malicious files to the number of total malicious files.

In this paper, we add a new metric, coverage, to measure how many testing data can be predicted by FILM, i.e., coverage =1−(number of unpredictable test files)/(number of test files). We exclude the unpredictable files when computing accuracy, precision, and recall for FILM; therefore, there is accuracy-coverage trade-off in FILM.

Table 3 summarizes the experimental results. Four base deep learning models are implemented and tested separately. For each model, the performance is measured twice when FILM is applied and when FILM is not applied. For example, the accuracy, precision, and recall of model 1 is 0.9788, 0.9869, and 0.9132, respectively. On the contrary, these metrics are all enhanced to 0.9936, 0.9967, and 0.9913, respectively, when FILM is applied; however, the coverage decreases to 0.9722. This means that FILM cannot predict 2.78% of test files, which can be uploaded to the cloud for further analysis. In return, the edge node can predict 97.22% of test files with the high accuracy of 0.9936. We emphasize that the accuracy of each model is different from each other. Model 3 shows the lowest accuracy among four models; however, its accuracy is also enhanced to 0.9879 with coverage of 0.6999 when FILM is applied.

We confirm that the experimental results show that FILM can enhance the accuracy, precision, and recall of all the four well-known ML models without any exception. The key innovation is that FILM can work with any model and enhance the existing model’s performance without increasing any time and space complexity. We emphasize that these four models are different from each other, but FILM enhances all model’s performance. The main idea of [11] motivates us to present FILM, but this paper is the first that classifies suspicious files into one of benign, malicious, and unpredictable. Those benign and malicious files of the similar static-analysis features are now classified as unpredictable instead of being incorrectly classified as benign or malicious. The experiments were performed with four existing ML models and real malware datasets.

Although FILM successfully enhances all of the accuracy, precision, and recall of existing ML models, there is a fundamental research challenge with learning-based malware detection. First, the learned model may not correctly predict the label of a suspicious file if the distribution of the test dataset becomes different from that of the training dataset, called concept drift [19]. Second, the deep learning model does not explain the reason why it considers the suspicious file as benign or malicious. In FILM, we simply use the sigmoid function result and confirm that the prediction is correct; however, there is no technique to depict which part of the suspicious file is the main reason for the decision. The future work of FILM would include these research challenges and we will extend it to other file types, such as Android APK files and office document files.

## 6. Conclusions

In this paper, we showed how edge computing can provide a ML-based malware detection service to IoT devices with sensors. We present a new decision method, FILM, for edge computing that can classify a suspicious file into benign, malicious, or unpredictable while state-of-the-art methods only make a binary decision about either a benign or malicious file. The idea is to append a sigmoid function to the last layer of any existing ML model and to interpret the function value as a confidence score. We implemented four well-known ML models and applied FILM to all of them. Through experiments on real malicious and benign file datasets, we confirmed that FILM enhanced all the accuracy, precision, and recall of the four models without any exception. Because FILM does not increase time and space complexity, this can be practically utilized. We leave the concept-drift problem of ML-based malware detection as a future work.

## Figures and Tables

**Table 1:** Summary of related work.

Ref.	ML Model	Dataset/Feature	Main Goal	Prediction Type
[5]	SVM, RF, NN	PE/static	Show ML limits on packed files	Benign vs. Malicious
[9]	NN	PE/static	Accurate malware detection	Benign vs. Malicious
[15]	SVM, RF, NN	APK/static	Show experimental bias of ML	Benign vs. Malicious
[16]	CNN	PE/static	Accurate malware detection	Family type
[17]	NN	PE/static	Accurate malware detection	Benign vs. Malicious
[18]	CNN, RNN	PE/dynamic	Accurate malware detection	Benign vs. Malicious

**Table 2:** Comparison of malware analysis methods in terms of time and cost.

Type	Method	Time	Cost
signature matching	string matching, regular expression	seconds or less	low
static analysis	file leader, strings disassembled code	seconds∼minutes	middle
dynamic analysis	virtual machine, sandbox	minutes∼hours	high
human analysis	manual analysis	hours∼days	very high

**Table 3:** Comparison of ML models with and without FILM.

Type	FILM(X)				FILM(O)			
	Accuracy	Precision	Recall	Coverage	Accuracy	Precision	Recall	Coverage
Model 1 [9]	0.9788	0.9869	0.9132	1.0000	0.9936	0.9967	0.9913	0.9722
Model 2 [16]	0.9679	0.9650	0.9754	1.0000	0.9954	0.9952	0.9962	0.9550
Model 3 [17]	0.9395	0.9296	0.9409	1.0000	0.9879	0.9878	0.9881	0.6999
Model 4 [18]	0.9798	0.9811	0.9810	1.0000	0.9906	0.9906	0.9917	0.9660

## Data Availability

The dataset includes malware files that may harm computer systems and therefore should not be open to public. Please contact the corresponding author for the dataset if you need it.
